# Multiple Sclerosis and MEN2 Neoplasia in a Female Patient: A Unique Co-Existence with Expanded Immunological Interest and Therapeutical Challenges, before and after Patient’s COVID-19 Infection

**DOI:** 10.3390/biomedicines10112850

**Published:** 2022-11-08

**Authors:** Nikolaos Markoglou, George Simeakis, Maria Alevizaki, Georgios Velonakis, Theofanis Chatzistamatiou, Maria Spyropoulou-Vlachou, Catherine Stavropoulos-Giokas, Leonidas Stefanis, Maria Anagnostouli

**Affiliations:** 1 Research Immunogenetics Laboratory, 1st Department of Neurology, Medical School, National and Kapodistrian University of Athens, Aeginition University Hospital, 11528 Athens, Greece; 2 Multiple Sclerosis and Demyelinating Diseases Unit, 1st Department of Neurology, Medical School, National and Kapodistrian University of Athens, Aeginition University Hospital, 11528 Athens, Greece; 3 Endocrine Unit, Department of Clinical Therapeutics, Alexandra Hospital, School of Medicine, National and Kapodistrian University of Athens, 11528 Athens, Greece; 4 Research Unit of Radiology, 2nd Department of Radiology, Medical School, National and Kapodistrian University of Athens, 11528 Athens, Greece; 5 Histocompatibility & Immunogenetics Laboratory, Hellenic Cord Blood Bank, Biomedical Research Foundation, Academy of Athens, 11528 Athens, Greece; 6 1st Department of Neurology, Medical School, National and Kapodistrian University of Athens, Aeginition University Hospital, 11528 Athens, Greece

**Keywords:** multiple sclerosis, pediatric-onset, MEN2, neoplasia, rare diseases, HLA, KIR, PD-1, immunopathogenesis, immunotherapeutics

## Abstract

Multiple sclerosis (MS) and its various comorbidities that may be observed are of great interest due to the complexity of MS pathophysiology and all of the immunological changes that follow. The incidence of cancer in MS has been investigated for several years, as not only does it affect ongoing therapeutical decisions, but also, certain disease-modifying treatments (DMTs) may increase the risk of tumorigenesis. For the first time, we present a case of a female patient with pediatric-onset MS (POMS) and multiple endocrine neoplasia 2B (MEN2B) and analyze the immunological impact of these diseases on the therapeutical choice, under the umbrella of her COVID-19 infection and the SARS-CoV-2 pandemic as a whole. We also review the existing literature regarding the immunogenetic and immunological correlations between these two extremely rare diseases and discuss the most suitable treatment for our case, which seems to be an anti-CD20 agent due to a better outcome in putative MS worsening and tumor progression, when killer immunoglobulin-like receptors’ (KIR) expression is reduced in natural killer (NK) cells. We also broaden our concerns on this comorbidity issue, at the same time focusing on the future research needed in this unexplored field of the comorbidity of MS and cancers.

## 1. Introduction

Multiple sclerosis (MS) is a chronic, inflammatory, demyelinating disease of the central nervous system (CNS), affecting mostly young adults at their reproductive age, adolescents and children [1]. The prevalence of pediatric-onset MS (POMS) is 3–5% of the total MS cases, and the strongest genetic correlation originates from HLA class II alleles on chromosome 6p21 and, particularly, the HLA DRB1*15:01 allele, both for pediatric- and adult-onset MS [2]. HLA analysis is being studied as a potential biomarker for the selection of certain therapeutic agents in MS, offering personalized treatment [3]. The most common comorbidities in MS are psychiatric, cardiovascular and other autoimmune disorders [2]. The prevalence of cancer as a comorbidity with MS has long been studied, and the findings are ambiguous. Certain HLA alleles have been associated with tumorigenesis, especially with breast cancer and nasopharyngeal cancer, providing a link between autoimmunity and cancer [4].

Multiple endocrine neoplasia (MEN) syndromes describe an autosomal dominant group of heterogeneous disorders, characterized by multiple endocrine tumors persisting in two or more endocrine glands. There are four types of the disease (MEN1-4), each one with a different clinical presentation and genetic burden [5] (Table 1).

When searching the immunogenetic correlation between MS and MEN2, we found a research gap, especially regarding the linkage between HLA and MEN2. On the other hand, a correlation between medullary thyroid carcinoma (MTC) and certain HLA alleles was observed. *HLA-DR7* and *HLA-DR2* have been described as risk factors in the manifestation of MTC, but studies have found no correlation between HLA and MTC as part of the MEN syndromes [6].

An exploration of the immune microenvironment of tumors, especially that of MTC, led to interesting findings. The absence of MHC I expression on the surface of tumor cells has been described, which results in tumor immune escape [7]. The lack of MHC I in tumor cells stimulates natural killer (NK) cells due to the absence of reaction between MHC I with KIR (killer immunoglobulin-like receptor), which are expressed on the surface of NK cells. KIR have great impact on the regulation of NK cells in the manifestation of cancer. When there is no reaction between KIR and MHC, NK cells are less sensitive when targeting tumor cells [8]. On the other hand, the reduced expression of MHC on the surface of tumor cells protects them from being detected by cytolytic T cells [8]. Several inhibitory molecules are expressed on the surface of tumor cells, such as PD-1 (programmed cell death protein 1), TIM-3 (T-cell immunoglobulin and mucin-domain containing-3), Lag-3 (Lymphocyte-activation gene 3) and TIGIT (T-cell immunoreceptor with immunoglobulin and ITIM domain). These molecules negatively affect the regulation of T cells by downregulating the production of inflammatory cytokines IL-2, TNF-α and IFNγ [7]. Regulatory T cells (Tregs) also provide tumor progression via the overexpression of immune inhibition molecules such as PD-1 and CTLA-4. Targeting against Treg would be a beneficial tumor therapeutic approach [6]. Due to these mechanisms of tumor cells escaping the immune system, research has focused on developing immunomodulatory therapies to achieve a better therapeutic approach [7].

This rare clinical case shows a patient presenting with a newly described comorbidity of MEN2 and POMS. This is the first case with a co-existence of these rare entities described in the literature. Due to the importance of HLA in the pathophysiology and treatment of immune-mediated diseases, such as MS and cancer [3], we tried to discover an association of certain HLA alleles with MS and MEN2B but found no linkage. On the other hand, HLA molecules play a crucial role in MTC pathogenesis, which is the most common cancer type in MEN2. The combined effectiveness of DMTs in both MS and MEN2B are described through the experience of our case, while the impact of DMTs in cancer in general is also reviewed. There is a general agreement that immunosuppressant DMTs for MS lead to elevated risk of cancer, while the role of immunomodulatory DMTs in cancer development is still under investigation [9]. However, after thorough review of the existing literature, we conclude that anti-CD20 agents are the best personalized therapeutic choice for this specific patient in case of worsening disease.

## 2. Case Illustration

A 28-year-old woman of Greek origin was referred to Aeginition University Hospital with dizziness, walking instability and double vision. Concerning her past neurological history, she reported a history of migraine that started at the age of 17 and an episode with blurred vision and dizziness three years prior, at 14 years of age, that lasted a few days. A brain MRI was performed that revealed three demyelinating lesions without enhancement. Due to the strong suspicion of pediatric-onset MS (POMS), cervical and thoracic MRI of the spinal cord and lumbar puncture were suggested, but the patient did not consent to these procedures. She and her family thought that all these symptoms could be due to the stressful period she was experiencing during the initial diagnosis of MTC and everything that followed. However, from time to time she had dizziness and double or blurred vision that would subside in few days. Diplopia was still her main fluctuating but persistent symptom.

**Figure 1:**
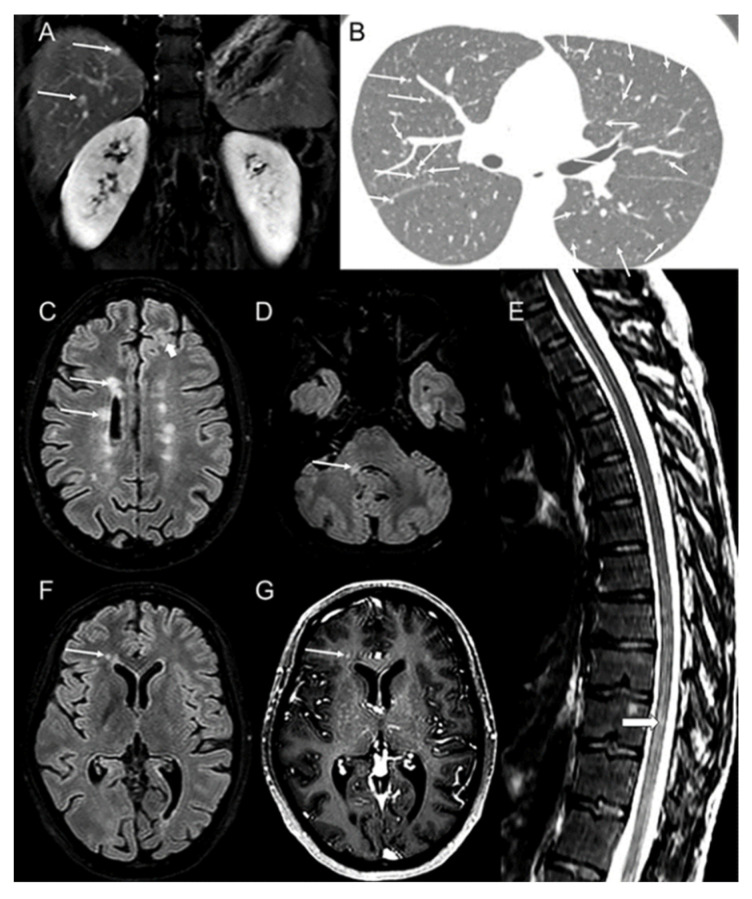
Abdominal MRI reveals hypervascular liver metastases (arrows (**A**)) from medullary thyroid carcinoma on coronal T1 post-contrast administration image (**A**). Chest CT (**B**) shows multiple micronodular lung metastases (arrows (**B**)). Brain MRI reveals multiple hyperintense and demyelinating lesions on axial FLAIR images (**C**,**D**) in the periventricular white matter (arrows (**C**)), in the subcortical white matter (thick arrow (**C**)), and in the right middle cerebellar peduncle (arrow (**D**)). There is also a lesion (thick arrow **E**)) in the thoracic spinal cord, as seen in the sagittal T2 image (**E**). One lesion seen on axial FLAIR image (**F**) near the frontal horn of the right lateral ventricle (arrows (**F**,**G**)) enhances axial T1 post-contrast image (**G**).

Regarding her general medical history, at the age of 15, she was referred to a gastroenterologist due to persistent diarrhea and loss of weight. During physical examination, a palpable neck mass was identified, and a thyroid ultrasound was performed that revealed nodules that may have been malignancies, as well as possibly infiltrated cervical lymph nodes. Basal calcitonin levels were at 1100 pg/mL and carcinoembryonic antigen (CEA) levels were at 256 ng/mL. The patient underwent total thyroidectomy with cervical lymphadenectomy, and the histopathological diagnosis of multifocal MTC with thyroid capsular and soft tissue invasion, as well as cervical lymph node infiltration, was established. After sequencing of the known exons of the *RET* gene involved in the pathogenesis of MTC, the patient was found to harbor an *RET* M918T–exon 16 mutation; thus, the case was defined as MEN2B. Two years later, and while calcitonin levels were elevated (4887 pg/mL), MRI revealed mediastinum as well as liver metastases (Figure 1A). The patient underwent liver chemoembolization, and treatment with a tyrosine kinase inhibitor (Motesanib) in the context of a clinical trial that was initiated. Disease stabilization, both structural and biochemical, was achieved for a period of six years when Motesanib administration was discontinued due to clinical trial interruption. Disease progression was observed during a period of three years, with new metastatic lesions in lungs (Figure 1B), liver and bones, while calcitonin and CEA levels were markedly elevated (45,017 pg/mL and 1234 ng/mL, respectively). Vandetanib, a multikinase inhibitor, was initiated achieving fair efficacy with structural and biochemical disease stabilization (calcitonin and CEA levels: 6643 pg/mL and 378 ng/mL, respectively) as well as good tolerability. The patient underwent Vandetanib treatment during the last 8 years with no exacerbations.

Her family medical record was negative for autoimmune diseases, MEN syndrome or MTC. Her father had a history of hypertension and diabetes type II. Her mother and sister had no health problems.

Her neurological examination on admission revealed cerebellar ataxia and anisocoria with positive photokinetic reflex. Brain MRI revealed periventricular, subcortical (Figure 1C) and infratentorial (Figure 1D) lesions with suspected demyelination. Lesions in the thoracic spinal cord were also observed on the spinal MRI (Figure 1E), as well as cerebral-enhancing lesions (Figure 1G). Based on MRI findings, both dissemination in space and time were established. The lumbar puncture was positive for type 2 oligoclonal bands, and the patient was diagnosed with multiple sclerosis since the 2017 McDonald criteria were fulfilled. In view of the established MS after her admission and regarding her past neurological history starting at puberty—with migraine and episodes of dizziness, blurred or double vision and the existence of demyelinating lesions in brain MRI—we had a strong suspicion of POMS. In our recent article on POMS, we presented that 18% of pediatric patients have diplopia as their first symptom [10] and other articles assure that migraine and diplopia are frequent first symptoms in POMS [11].

At this point, the patient did not start any medication. Two years later, she showed symptoms of dysarthria and Bell’s palsy and started receiving glatiramer acetate (Copaxone). For the following two years, she had exacerbations with optic neuritis, which subsided after receiving i.v glucocorticoids. Three years later, her treatment was altered to Teriflunomide (Aubagio).

Ten months after the initiation of Teriflunomide for MS, she tested positive for SARS-CoV-2. There was no abnormality in her blood exams and the absolute number of lymphocytes before and after the infection was normal (Table 2), although a series of concerns have emerged during the COVID-19 pandemic period [12]. One month later, she reported reduced visual acuity. The ophthalmologist’s exam was normal, while brain MRI revealed one new demyelinating lesion without gadolinium enhancement.

Her persistent symptoms are fluctuating blurred vision, numbness on the left lower lip and urgency to urinate. Her last clinical examination revealed a reduction in the visual acuity of the right eye, a positive Romberg’s test, difficulty in performing heel-to-toe movements and lower sensation in the left lower limb in comparison to the right. There were no pyramidal, brainstem and cerebral abnormalities. EDSS: 2.0.

### Next Generation Sequencing HLA Genotyping

For the HLA typing, peripheral blood samples (8 mL peripheral blood) were obtained by patients in sodium citrate (ACD Vacutainer^®^, Becton Dickinson, Vacutainer Glass ACD Solution Tube, NJ, USA) and samples were processed, as described elsewhere [13]. DNA extraction and further NGS analysis were carried out in the Histocompatibility and Immunogenetics Laboratory, Biomedical Research Foundation, Academy of Athens, Greece, with the Holotype HLA 7/96 CE v2 kit (Omixon, Budapest, Hungary), as per the manufacturer’s instructions [13]. The patient’s NGS-HLA genotyping is described in Table 3.

## 3. Discussion

In this paper, we present a patient with an exceptional comorbidity of two rare or/and extremely rare diseases, POMS and MEN2B, and to our knowledge, this is the first such case in the literature. No genetic or immunogenetic correlation between the two diseases has been discovered thus far. Our patient’s immunogenetic profile revealed the presence of HLA-DQA1*01:02, DQB1*06:02, and DRB1*15:01, which are risk factors for MS, while she is homozygote for the protective allele for MS, HLA-DPB1*04:01, regarding the Hellenic population [9]. On the other hand, the immunogenetic risk factors for MTC, which is the main manifestation of MEN2B, such as *HLA-DR2* and *DR7*, were not present in our patient. Of course, one has to note that, in the case of MEN2B, cancer has an inherited genetic etiology with a high penetrance, although immunogenetic background always plays a core role, both for autoimmune and malignant diseases. As described above, tumor cells of MTC escape from the immune system, a mechanism that makes treatment more complex.

Recently, the benefit of using DMTs for both MS and cancer incidence has been researched. On the other hand, the mechanism of DMT-associated cancer may be the decreased migration of T cells to tumor sites, which contributes to decreased cytotoxicity against tumor cells. Immunomodulatory therapies that can restore the ability of immune cells against tumor cells would be beneficial for both MEN2B and MS. The question that arises from our case is the possibility of taking advantage of the immunomodulatory treatment given for MS in order to have therapeutic advantages in MEN2B. The immunological changes due to the pandemic of SARS-CoV-2 should also be evaluated for the appropriate treatment. After SARS-CoV-2 infection, a decrease in lymphocytes was observed, especially in CD4^+^ T-cells, CD8^+^ T cells, B cells and NK cells. Immunophenotyping analyses showed a decrease in both memory T cells (CD3^+^CD4^+^CD45RO^+^) and CD3^+^CD8^+^CD28^+^ cytotoxic T cells. The observed lymphopenia is thought to be dependent on the severity of the SARS-CoV-2 infection [14]. There were no other immunophenotype evaluations, all from previous years, to use for reference, even if the patient was under certain immunotherapies for both diseases. After her mild COVID-19, we observed no abnormal values of total CD4 and CD8 T lymphocytes, while a decrease in central CD4 and CD8 lymphocytes was noticed. The NK cells had normal values one month after SARS-CoV-2 infection, while a reduction in the absolute number was observed 6 months after the infection. (Table 2).

There is no used DMT, for MS, targeting specific molecules on the surface of tumor cells or the respective receptors on the surface of T cells. Due to the above-mentioned mechanisms of the immune escape of tumor cells, certain therapeutic agents should be used, targeting the right molecules to increase antitumor effects. The majority of DMTs in MS are factors that have primarily been used in rheumatologic autoimmune diseases and hematologic malignancies. By reviewing the already existing DMTs for MS and their antitumor effects, useful information can be deduced (Table 4).

Interferons, especially IFN-β, which is used in MS, have antitumor efficacy [15] in malignancies, such as neuroblastoma, melanoma, breast cancer and cervical cancer. On the other hand, cases of breast cancer in patients receiving IFN-β have been described [16].

Glatiramer acetate (GA) is a well-tolerated DMT used in RRMS patients with low EDSS progression that targets both the innate and adaptive immune system, with rare cases of tumorigenesis having being described [17].

Dimethyl fumarate (DMF) is a DMT that is initially used in psoriasis and in MS with great effects [18]. Antitumor effects have been described, while the incidence of malignancies as side effect is notable.

Teriflunomide, which our patient receives, has several antitumor effects, but none of the known effects are related to the growing mechanism of MTC [19]. Cases of tumorigenesis have been described [20].

Fingolimod as a sphingosine-1-phosphate receptor modulator sequesters lymphocytes in lymph nodes, preventing them from contributing to an autoimmune reaction and, in some studies, has been shown to have antitumor actions. It may cause immunosuppression against Treg cells that contribute to the tolerance of malignant tumor cells [17]. On the other hand, tumorigenesis has been described in patients receiving Fingolimod [21].

Natalizumab had no successful effects as antitumor treatment, while cases of breast cancer, melanoma and diffused B-cell lymphoma have been described [22].

Cladribine was firstly investigated as an effective treatment for leukemias but was soon rejected due to adverse events [23]. The high prevalence of several types of cancer in trials using Cladribine as DMT in MS patients makes it a risky treatment for our patient.

Anti-B cell therapies, such as Rituximab and Ocrelizumab, are highly effective in MS patients, reducing EDSS progression and the demyelinating activity. Those monoclonal antibodies have been used in the treatment of leukemias, as the CD20 cell surface is found in most tumor cells [24]. Despite the great action as antitumor and immunosuppressant therapy, several cases of malignancy have been described [17].

Tyrosine kinase inhibitors have been suggested as effective complementary therapies in MEN2B and the treatment of our patient, Vandetanib, is a multi-kinase inhibitor targeting VEGFR, RET and MET [25]. In phase III clinical trials for MS, Bruton’s tyrosine kinase inhibitors (BTKis) are being performed and currently demonstrate promising results. It is well known that BTKis are already used for the treatment of lymphomas and leukemias, but their action in solid tumors is not well established [25].

Although our patient has received Teriflunomide in the last few years, which is correlated with the development and progression of some types of cancer, no exacerbation of her disease has been observed. On the other hand, careful decisions about the next therapeutic choices for MS should be made regarding the possibility of MS exacerbation. From the above-mentioned information, anti-B cell therapies seem to be the most suitable choice for both diseases, with maximum effectiveness and the minimum possibility of MEN2B exacerbation. Rituximab enhances the antibody-dependent cellular cytotoxicity (ADCC) of NK cells through the interaction between the CD-16 activating receptor of NK cells with Fc fragments of anti-CD20 monoclonal antibody. The interaction between KIR of NK cells with MHC I of tumor cells results in a reduced antitumor efficacy in Rituximab [26]. As a result, research shows a better outcome of anti-CD 20 treatment for tumor progression, when KIR expression is reduced [26].

## 4. Conclusions

In conclusion, the comorbidity of MS with cancer has been thoroughly researched in recent years. Additionally, therapies for patients with these coexisting conditions have been suggested; however, unanswered questions remain. We tried to explain the coexistence of MS and MEN2B through common immunological and immunogenetic pathways, but no HLA linkage was found in the literature and in our case. Nevertheless, there are exceptional cases, such as our patient, that need personalized treatment because of the coexistence of two rare diseases, both with contrary immunological aspects. Considering the DMTs for MS that are already approved by the FDA, there is no ideal option that we could suggest as a combined therapy using the currently approved DMTs. Even though anti-B cell therapies seem to be the most appropriate therapeutic agents for this unique patient, in case of the MS disease worsening, without having a negative impact on MEN2B, more specific research into diverse comorbidities is needed in this field. This research field attracts great interest from the field of immunology, and future research should be conducted given the new immunological and non-immunological parameters of the COVID-19 pandemic, which must be taken into serious and urgent consideration.

## Figures and Tables

**Table 1:** Clinical characteristics and epidemiology of MEN syndromes.

Type	Clinical Characteristics	Prevalence	Genetic Burden
MEN1	Affects the parathyroid gland, the pancreas and the pituitary gland	1:30,000	Mutation in the MEN1 gene
MEN2A	Medullary thyroid carcinoma (MTC), phaeochromocytoma (PC), hyperparathyroidism (HPT)	1:25,000	Mutations in the RET
**MEN2B**	**MTC, PC, diffuse ganglioneuromatosis of the gastrointestinal tract**	**1:600,000**	**Mutations in the RET**
MEN3	MTC	0,2:100,000	Mutations in the RET
MEN4	Parathyroid tumors, anterior pituitary tumors in association with tumors of the kidneys, adrenals and reproductive organs	Unknown	Mutations in CDNK1B

**Table 2:** Lymphocyte immunophenotyping and absolute lymphocyte count at various timepoints.

Timepoint	WBC	ALC	CD4	CD8	CD19	NK
One month before SARS-CoV-2 infection–Teriflunomide(+)	9780/μL	1600/μL N.V: (1200–3800)				
During SARS-CoV-2 infection–Teriflunomide(−)	11230/μL	1627/μL				
One month after infection/ Before re-initiation of Teriflunomide	10490/μL	1476/μL N.V: (1200–3800)	705/μL N.V:(489–1666)	239/μL N.V: (188–1082)	158/μL N.V: (47–493)	205/μL N.V: (145–678)
Six months after infection/ After re-initiation of Teriflunomide	8610/μL	1717/μL N.V: (1200–3800)	968/μL N.V: (489–1666)	281/μL N.V: (188–1082)	240/μL N.V: (47–493)	120/μL N.V: (145–678)

WBC: white blood cell; ALC: absolute lymphocyte count; N.V: normal values.

**Table 3:** Patient’s NGS-HLA Genotyping.

Sample	Allele	HLA-A	HLA-B	HLA-C	HLA-DPB1	HLA-DQA1	HLA-DQB1	HLA-DRB1
	**Allele 1**	A*33:03:01	B*18:01:01	C*12:03:01	DPB1*04:01:01	DQA1*01:02:01	DQB1*03:01:01	DRB1*11:04:01
	**Allele2**	A*68:01:02	B*41:01:01	C*17:01:01	DPB1*04:02:01	DQA1*05:01:01	DQB1*06:02:01	DRB1*15:01:01

* the international and official expression of HLA alleles.

**Table 4:** DMTs function in MS and correlation with cancer.

DMTs	Immunologic Function in MS	Anti-Tumor Effects	Cancer Type Correlated with
IFNs	Decrease in antigen presentation and change in the expression of Th1 lymphocytes	Inhibition of tumor cell differentiation and proliferation	Breast
GA	Blockage of MHC-II * in immunological synapsis and shift from Th1 to Th2 immune responses	Upregulation of anti-inflammatory M2 monocytes, Th2 cells and T regulatory cells (Tregs)	Breast, skin
DMF	Antioxidant properties and reduction in inflammatory molecules	Apoptosis of tumor cells	Breast, basal cell carcinoma
Teriflunomide	Inhibition of the proliferation of autoreactive B and T cells	Downregulation of anti-apoptotic proteins in tumor cells	Cervical
Fingolimod	Entrapment of T lymphocytes in lymph nodes. Reduction in circulating lymphocytes in the peripheral blood	Immunosuppression against Treg cells that contribute to tolerance of malignant tumor cells	Basal cell carcinoma, breast, thyroid
Natalizumab	Inhibition of the migration of lymphocytes through the BBB *	Blocks cell adhesion	Breast, melanoma, diffused B-cell lymphoma
Cladribine	Depletion of T and B lymphocytes	Chain termination and cell-death	Melanoma, ovarian, pancreatic
Anti-B cell	Depletion of B lymphocytes	Promotes tumor cell-death	Renal, melanoma, breast

* MHC: major histocompatibility complex, BBB: blood–brain barrier.

## Data Availability

Data is contained within the article.

## Abbreviations

MS	multiple sclerosis
POMS	pediatric-onset multiple sclerosis
RRMS	relapsing remitting multiple sclerosis
CNS	central nervous system
HLA	human leucocyte antigen
MHC	major histocompatibility complex
DMTs	disease-modifying treatments
MEN	multiple endocrine neoplasia
MTC	medullary thyroid carcinoma
PC	phaeochromocytoma
HPT	hyperparathyroidism
RET	rearranged during transfection
NK	natural killer
WBC	white blood cell
Tregs	T regulatory cells
KIR	killer Immunoglobulin-like receptors
PD-1	programmed cell death protein 1
TIM-3	T-cell immunoglobulin and mucin-domain containing-3
Lag-3	lymphocyte-activation gene 3
TIGIT	T-cell immunoreceptor with immunoglobulin and ITIM domain
